# miR-212-3p attenuates neuroinflammation of rats with Alzheimer’s disease via regulating the SP1/BACE1/NLRP3/Caspase-1 signaling pathway

**DOI:** 10.17305/bjbms.2021.6723

**Published:** 2022-02-12

**Authors:** Wei Nong, Chuanhong Bao, Yixin Chen, Zhiquan Wei

**Affiliations:** Guangxi University of Chinese Medicine, Nanning, Guangxi, China

**Keywords:** Alzheimer’s disease, miR-212-3p, SP1, BACE1, NLRP3/Caspase-1 signaling pathway, pyroptosis, transcription factor, neuroinflammation, Aβ1-42, pathological injury

## Abstract

Alzheimer’s disease (AD) ranks as the leading cause of dementia. MicroRNA (miR)-212-3p has been identified to exert neuroprotective effects on brain disorders. The current study analyzed the protective role of miR-212-3p in AD rats through regulating the nucleotide-binding oligomerization domain-like receptor family pyrin domain-containing 3 (NLRP3)/Caspase-1 signaling pathway. The AD rat model was established through injection of amyloid-β 1-42 (Aβ1-42), followed by the Morris water maze test. The morphology and functions of neurons were observed. Furthermore, miR-212-3p, NLRP3, cleaved Caspase-1, gasdermin D N-terminus, interleukin (IL)-1β, and IL-18 expressions were measured. H19-7 cells were treated with Aβ1-42 to establish the AD cell model, followed by an assessment of cell viability and pyroptosis. Downstream targets of miR-212-3p and specificity protein 1 (SP1), as well as beta-site amyloid precursor protein cleaving enzyme 1 (BACE1), were predicted by databases and testified using dual-luciferase and chromatin immunoprecipitation assays. miR-212-3p was weakly expressed in AD rats. miR-212-3p overexpression was linked to improved learning and memory capacities of AD rats and reduced neuronal pyroptosis linked to neuroinflammation attenuation. In vitro, miR-212-3p improved viability and suppressed pyroptosis of neurons through inhibiting NLRP3/Caspase-1. Overall, miR-212-3p inhibited SP1 expression to block BACE1-induced activation of NLRP3/Caspase-1, thereby attenuating neuroinflammation of AD rats.

## INTRODUCTION

Alzheimer’s disease (AD), the major subtype of dementia, is known as a progressive neurodegenerative disease in the elderly [1]. AD is predominately associated with accumulation of amyloid-beta (Aβ), hyperphosphorylation of tau protein, increased oxidative stress and mitochondria dysfunction, as well as environmental risks, leading to loss of synapses, neurons cognitive impairment, and even death at the late stage [2-4]. Neuroinflammation is a microglia-activated pathological process to eliminate toxic components in the central nervous system (CNS) [5]. Neuroinflammation process in AD, which is a response to the generation of Aβ, hyperphosphorylated tau protein, and oxidative molecules, is important to be resolved to alleviate the symptoms of the disease [6,7]. Unfortunately, only a few molecules targeting neuroinflammation displayed effects convincing enough to support their usage in clinical treatment [5]. In this context, the discovery of novel molecules with conclusive efficacy in neuroinflammation may confer a glimmer of hope for the AD management.

Pyroptosis, an inflammatory and lytic cell death, could elicit and spread neuroinflammation in neurodegenerative diseases [8]. Nucleotide-binding oligomerization domain-like receptor family pyrin domain-containing 3 (NLRP3) inflammasome, functioning as the promoter of the innate immune system, is characterized by NLRP3/Caspase-1 activation, and its inhibition exerts neuroprotective effects in AD through restricting neuronal pyroptosis [9,10]. Furthermore, NLRP3/Caspase-1 signaling pathway has been demonstrated to affect the development of AD through regulating neuroinflammation [11]. Therefore, targeting the NLRP3/Caspase-1 axis is beneficial to inhibit neuronal pyroptosis, followed by the neuroinflammation attenuation.

MicroRNAs (miRNAs), a class of short non-coding RNA normally with 22-23 nucleotides, are altered along with mitochondrial impairment, oxidative imbalance, tau phosphorylation, and neuron damage, and in turn, dysfunctional miRNAs continue to aggravate neuroinflammation and cognitive impairment [12-14]. miR-212 is essential for the normal function of mammalian CNS and its dysregulation results in the occurrence of neurodegenerative and neurocognitive diseases, including AD [15]. Inherently, miR-212-3p overexpression is capable of attenuating Aβ-induced neurotoxicity and neuronal injury in AD [16,17]. Nevertheless, the crosstalk of miR-212-3p and the NLRP3/Caspase-1 signaling pathway in AD has not been researched before.

In terms of its downstream mechanism, miR-212-3p was previously demonstrated to target specificity protein 1 (SP1) in breast cancer [18]. SP1, from the category of the zinc finger protein family, acts as an inducer of inflammation and neuronal death [19]. Notably, SP1 triggers tau phosphorylation and Aβ-induced neuroinflammation [20,21]. Moreover, SP1 functions as a transcription factor to enhance the expression of its downstream targets, including beta-site amyloid precursor protein cleaving enzyme 1 (BACE1), thus participating in AD [22]. BACE1, a transmembrane protein in the pepsin family, is essential for the production of bioactive Aβ, initiating neurotoxicity and synaptic plasticity deficit in AD [23]. However, the regulatory relationship of miR-212-3p and the SP1/BACE1 axis in AD remains elusive.

Based on the aforementioned data, we hypothesized that miR-212-3p regulates the NLRP3/Caspase-1 signaling pathway through suppression of the SP1/BACE1 axis to attenuate neuroinflammation in AD. Therefore, we established the AD rat model and *in vitro* cell model to validate the role of miR-212-3p as a therapeutic target in AD treatment.

## MATERIALS AND METHODS

### AD rat model establishment

Specific pathogen-free level Sprague-Dawley male rats (8 weeks, 250 ± 20 g) (Hunan SJA Laboratory Animal Co., Ltd, Hunan, China) were raised under conditions of 20-25 °C, 60-65% humidity, and 12 hours day/night cycles and had unrestricted access to food and water. After rats were trained for 2 weeks, the Morris water maze test (MWMT) was performed to exclude rats with congenital dementia or adverse swimming stroke.

After weighing, rats were anesthetized with an intraperitoneal injection of pentobarbital sodium (30 g/kg). According to the brain stereotaxic atlas [25], two holes with a diameter of 1 mm on the rat’s skull (3.3 mm behind anterior fontanelle and 1.5 mm from the middle line) were drilled. Then, amyloid-β 1-42 (Aβ1-42) (5 μL, 100 μM, Abcam, Cambridge, MA, USA) was injected into the hippocampus (depth: 3.5 mm) using a microinjector within 5 minutes at the rate of 1 μL/minute, and the needle was pulled out at 1.0 mm/minute. After the holes were sealed with resin powder, the scalp was sutured and externally applied by penicillin powder, and rats were intramuscularly injected with penicillin after the surgery (once a day, for 3 consecutive days, Sigma, St. Louis, MO, USA) to prevent infection. Rats in the sham group (n = 6) were intracranially injected with the same volume of normal saline and were given the same treatments as the model rats [26].

### Animal grouping and treatment

The 18 model rats were randomized into three groups: AD group, AD + agomir NC group, and AD+miR-212-3p agomir group. At 24 hours after AD induction, the third ventricle [26] was injected with 50 μL agomir NC or miR-212-3p agomir (1 nmol/50 μL, Guangzhou RiboBio Co., Ltd., Guangdong, China) [27,28].

### MWMT

On the 1^st^ day after 4 weeks of treatment, MWMT was performed to assess rat spatial learning and memory capacities using a round tank (diameter: 214 cm, height: 50 cm, Noldus (Beijing) Information Technology Co., Ltd., Beijing, China) with a camera above the tank for a full view of the channel. The experiment was performed in a quiet room with artificial lighting. The water (20-23 °C, 30 cm) was changed every day.

Rats fasted for 4 hours before the experiment. MWM was divided into four quadrants: I, II, III and IV, where a platform was placed 2 cm above the water in quadrant I. Each rat was trained 4 times a day for 5 consecutive days. Rats were trained to swim to the platform from different sites in quadrants II, III, and IV and stay on it for 20 seconds. The escape latency referred to the time that rats took to seek the platform and was calculated as the mean value of tests each day. Time longer than 120 seconds was recorded as 120 seconds. On the 6^th^ day, the directional navigation test was performed. On the 7^th^ day, the space probe test was conducted and the platform was removed from MWM. The time spent in the target quadrant and the number of platform crossing with 90 seconds was recorded. All data were automatically recorded using SuperMaze software.

### Hematoxylin and eosin (H&E) staining

After MWMT, rats were euthanized using excessive pentobarbital sodium (100 mg/kg, i.p.). Rat’s brain tissues were perfused using 500 mL mixture of 2% paraformaldehyde (Sigma-Aldrich, St. Louis, MO, USA) and 2% polyglutaraldehyde (Sigma-Aldrich) at 4 °C for 2 hours. After that, the hippocampus was extracted at 4 °C and fixed in the mixture devoid of light for 12 hours. On the next day, tissues were embedded and sliced into 4 mm thick sections. Thereafter, hippocampus sections were treated with H&E staining for the observation of neuronal morphology. Briefly, sections were heated at 60 °C overnight, dewaxed in xylene I and xylene II successively for 20 minutes, and left in gradient ethyl alcohol (100%, 100%, 95%, 80%, and 70%), respectively, for 5 minutes. Then, dewaxed sections were washed with distilled water and stained with hematoxylin (Sigma-Aldrich) for 10 minutes. Following, sections were rinsed with tap water for 15 minutes, stained with eosin (Sigma-Aldrich) for 30 seconds, dehydrated, and mounted.

### Nissl’s staining

Sections of the hippocampus were immersed in 100%, 95%, 80%, and 70% xylene respectively for 2 minutes and stained with 1% toluidine blue (Sigma-Aldrich) at 56 °C for 40 minutes. Then, stained sections were rinsed with running water for 8 minutes, dehydrated in gradient ethyl alcohol (70%, 80%, 95%, and 100%) for 2 minutes, cleaned with xylene I and xylene II, respectively, for 3 minutes, and sealed using neutral glue. After that, the number of Nissl-positive cells was counted under a microscope [28].

### Cell culture and Aβ 1-42 induction

Immortalized rat hippocampal neuron cell line H19-7 (Shanghai Beinuo Biotechnology, Shanghai, China) was cultured in Dulbecco’s modified Eagle’s medium containing 10% fetal bovine serum, 1% penicillin, and 1% streptomycin (Gibco, Thermo Fisher Scientific, Waltham, MA, USA) at 37 °C with 5% CO_2_. The culture medium was changed every 2 days. H19-7 cells were seeded to 96-well plates and remained equilibrated for 24 hours before subsequent experiments. Aβ1-42 (Sigma-Aldrich, St. Louis, MO, USA) (dissolved in deionized water at 100 mM concentration) was diluted to the desired concentration before usage. During AD cell model establishment, H19-7 cells were induced with 10 mM Aβ1-42 diluent for 24 hours [29,30].

### Cell transfection

miR-212-3p mimic, SP1 overexpression pcDNA-SP1 plasmid (oe-SP1), BACE1 overexpression pcDNA-BACE1 (oe-BACE1), and their corresponding controls (empty vectors, oe-NC) were provided by GenePharma (Shanghai, China). Cell transfection was carried out strictly following the instructions of lipofectamine 2000 (Invitrogen, Carlsbad, CA, USA). After 48 hours, transfected cells were induced with 10 mM Aβ1-42 diluent for the following experiments [31].

### Reverse transcription quantitative polymerase chain reaction (qRT-PCR)

Being extracted by the RNeasy Mini kit (Qiagen, Valencia, CA, USA), the total RNA was reverse transcribed using the reverse transcription kit (RR047A, Takara, Tokyo, Japan) to harvest the complementary DNA (cDNA). In terms of miRNA detection, miRNA was reverse transcribed into cDNA with application of the miRNA first-strand cDNA synthesis (Tailing Reaction) kit (B532451-0020, Sangon, Shanghai, China). qRT-PCR was processed using the SYBR® Premix Ex TaqTM II (Perfect Real Time) kit (DRR081, Takara) and real-time quantitative PCR analyzer (ABI 7500, ABI, Foster City, CA, USA). The amplification procedures included two steps: The first step was pre-denaturation at 95 °C for 30 seconds, and the second step was 40 cycles of 95 °C for 5 seconds and 60 °C for 34 seconds. Each sample was equipped with three duplicate wells. qPCR primers were provided by Shanghai Sangon (primers sequences shown in Table 1). Ct value of each well was recorded and the relative expression of the product was calculated using the 2-ΔΔCt methods with β-actin or U6 as the internal reference. ΔΔCt = (mean Ct value of target genes from the experiment group - mean Ct value of housekeeping genes from the experiment group) - (mean Ct value of target genes from the control group - mean Ct value from housekeeping genes of the control group).

**TABLE 1:**
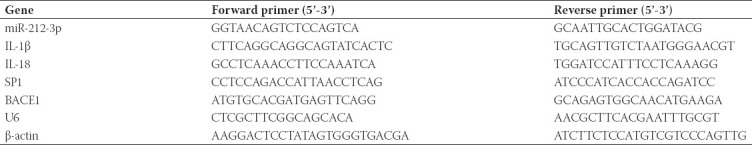
qPCR primers

### Western blotting

The enhanced RIPA lysis reagent (Boster Biological Technology Co. Ltd., Wuhan, China) containing the protease inhibitor was employed to lyse tissues and cells. Protein concentration was measured using the bicinchoninic acid protein quantification kit (Boster Biological Technology Co. Ltd.). Proteins to be tested were isolated by 10% sodium dodecyl sulfate polyacrylamide gel electrophoresis and electrically transferred to polyvinylidene fluoride membranes. Membranes were sealed with 5% bovine serum albumin at room temperature for 2 hours to block the non-specific binding, followed by the addition of diluted primary antibodies rabbit anti-β-actin (ab8227, 1:2000, Abcam), NLRP3 (ab263899, 1:1000, Abcam), GSDMD-N (ab215203, 1:1000, Abcam), cleaved Caspase-1 (#4199, 1:5000, Cell Signaling Technology, Shanghai, China), and mouse anti-SP1 (sc-17824, 1: 500, Santa Cruz Biotechnology, Dallas, Texas, USA) at 4 °C overnight. After washing, membranes were cultured with the secondary horseradish peroxidase-labeled goat anti-rabbit IgG (ab205718, 1:2000, Abcam) or goat anti-mouse IgG (ab6789, 1:2000, Abcam). The enhanced chemiluminescence working solution (EMD Millipore, Billerica, MA, USA) was used to capture Western blot images. The gray level of each band in Western blot images was quantified using Image-Pro Plus 6.0 (Media Cybernetics, San Diego, CA, USA) with β-actin as the internal reference. Each experiment was performed in triplicate.

### Cell viability

H19-7 cells of each treatment group were resuspended and seeded into 96-well plates at the density of 5 × 10^4^ cells/100 µL/well. Cells were treated with 10 μM Aβ1-42 for 24 hours. Subsequently, each well was added with methyl thiazolyl tetrazolium (MTT) (20 μL, 5 mg/Ml, [Sigma-Aldrich]) for 4 hours, followed by the addition of dimethyl sulfoxide solution (Sigma-Aldrich) to dissolve formazan crystals. The absorbance at a wavelength of 490 nm was determined using a microplate reader (Thermo Fisher Scientific).

### Enzyme-linked immunosorbent assay

Tissue homogenate was prepared by grinding on ice, followed by a centrifugation at 301 g for 10 minutes to harvest the liquid supernatant. H19-7 cells of each group were collected and washed with phosphate buffer saline (PBS) 3 times. On centrifugation at 100 g for 5 minutes, cells were incubated with PBS containing cell lysate at 4 °C for 10 minutes. After another round of centrifugation at 1204 g for 5 minutes, the contents of inflammatory cytokines (interleukin [IL]-1β and IL-18) in supernatant samples were determined using the ELISA kits (Cusabio Biotech Co., Ltd., Wuhan, China).

### Dual-luciferase reporter assay

The binding sites of miR-212-3p and SP1 were predicted with the help of the Jefferson website (https://cm.jefferson.edu/rna22/Interactive/RNA22Controller). SP1 3’UTR fragments containing wild and mutant-binding sites of miR-212-3p were inserted into pMIR-reporter plasmids (Beijing Hua Yueyang Biotechnology, Beijing, China) to construct SP1-WT and SP1-MUT plasmids. The above-constructed plasmids, mimic NC, or miR-212-3p mimic were cotransfected into H19-7 cells. After 48 hours, cells were harvested and lysed, before the measurement of luciferase activity using the luciferase detection kits (K801-200, BioVision, Mountain View, CA, USA).

The potential binding sites of SP1 and the BACE1 promoter which were analyzed through the JASPAR website (http://jaspar.genereg.net/) were employed to construct recombinant luciferase reporter gene vectors BACE1-WT and BACE1-MUT. The luciferase reporter gene vectors and oe-SP1 vector were cotransfected into H19-7 cells, followed by a dual-luciferase reporter assay to testify whether SP1 binds to the BACE1 promoter region. After 48 hours, cells were harvested and lysed, and luciferase activity was detected.

### Chromatin immunoprecipitation (ChIP)

Cells were fixed with formaldehyde to produce DNA-protein crosslinking. Then, an ultrasonic cell disruptor (10 seconds each time, 10 seconds interval, 15 cycles) was utilized to break cells and break chromatin into fragments. After that, cell debris was incubated with IgG antibody (ab172730, 1:1000, Abcam) and SP1 target protein-specific antibody (sc-17824, Santa Cruz Biotechnology) at 4 °C overnight. The DNA-protein compound was precipitated using Protein Agarose/Sepharose, followed by a centrifugation at 12,000 g for 5 minutes. The supernatant was discarded and the non-specific complex was washed. Next, the crosslinking was dismantled at 65 °C overnight, and DNA was extracted, purified, and recycled using the phenol-chloroform method. The binding of SP1- and BACE1-specific primer was detected through qRT-PCR.

### Ethical statement

This study was approved by the Ethics Committee of Guangxi University of Chinese Medicine. All animal experiments followed the Guide for the Care and Use of Laboratory Animals [24].

### Statistical analysis

Data statistical analysis and graphing were performed using SPSS21.0 statistical software (IBM Corp, Armonk, NY, USA) and GraphPad Prism 9.0 software (GraphPad Software Inc., San Diego, CA, USA). Measurement data were formalized as a mean ± standard deviation and were in line with normal distribution and homogeneity of variance. The t-test was employed for pairwise comparisons, one-way or two-way analysis of variance (was employed for multigroup comparisons, and Tukey’s multiple comparison test was employed for post-test. *p* value was obtained from two-sided tests. *p* < 0.05 meant statistical significance.

## RESULTS

### miR-212-3p was weakly expressed in AD rats and miR-212-3p overexpression improved neurological functions of AD rats

**FIGURE 1:**
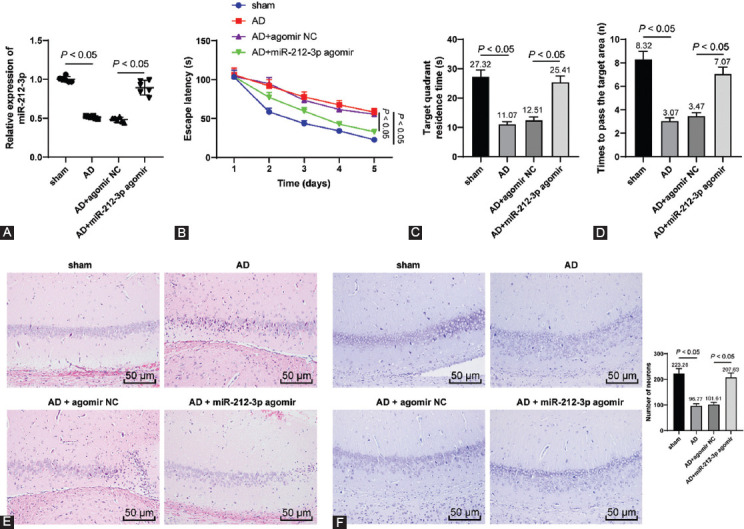
miR-212-3p was weakly expressed in AD rats and miR-212-3p overexpression improved neurological functions of AD rats. The AD rat model was established through injection of Aβ1-42, followed by the treatment of miR-212-3p agomir. (A) miR-212-3p expression in the hippocampus was detected through RT-qPCR; (B-D) animal’s learning and memory capacities were assessed through MWMT; (E and F) morphology and functions of neurons were observed through H&E staining (E) and Nissl’s staining (F). N=6. Data were formalized as mean ± SD. Data in Figures A and C-F were analyzed using one-way ANOVA, and data in Figure B were analyzed using two-way ANOVA, followed by Tukey’s multiple comparison test.

Recent research has illustrated that miR-212-3p was weakly expressed in AD [32,33]. However, the mechanism of miR-212-3p in AD remains unknown. To explore the functional mechanism of miR-212-3p in AD, the AD rat model was established using the treatment of Aβ1-42, followed by the injection of miR-212-3p agomir. It was found that miR-212-3p expression in the hippocampus was decreased in AD rats and overexpressed on the injection of miR-212-3p agomir (*p* < 0.05, Figure 1A). Further, the results of MWMT showed that, in comparison to the sham group, escape latency was markedly prolonged and the time in the target quadrant as well as times to pass the target area were markedly shortened in the AD group. On miR-212-3p overexpression, escape latency was markedly shortened, while time in the target quadrant and time to pass the target area were markedly prolonged (*p* < 0.05, Figure 1B-D). These results suggested that miR-212-3p could alleviate the learning and memory capacities of AD rats.

Next, the morphology of neurons in the hippocampus was observed through H&E staining. It was observed that the hippocampus of AD rats was presented with the disorder of neuronal layer, severe injury of neurons, pyknosis, and cytoplasm reduction. After miR-212-3p overexpression, the hippocampal CA1 region was presented with clear neuronal layers and an increased number of normal neurons with round nucleus (*p* < 0.05, Figure 1E). In addition, the results of Nissl’s staining showed that the number of Nissl body neurons was decreased in the hippocampus of AD rats, whereas miR-212-3p overexpression augmented the number of Nissl-positive neurons (*p* < 0.05, Figure 1F). The above results suggested that miR-212-3p was weakly expressed in AD rats and miR-212-3p overexpression could improve neurological functions of AD rats.

### miR-212-3p overexpression suppressed NLRP3/Caspase-1-activated neuronal pyroptosis to attenuate neuroinflammation in AD rats

**FIGURE 2:**
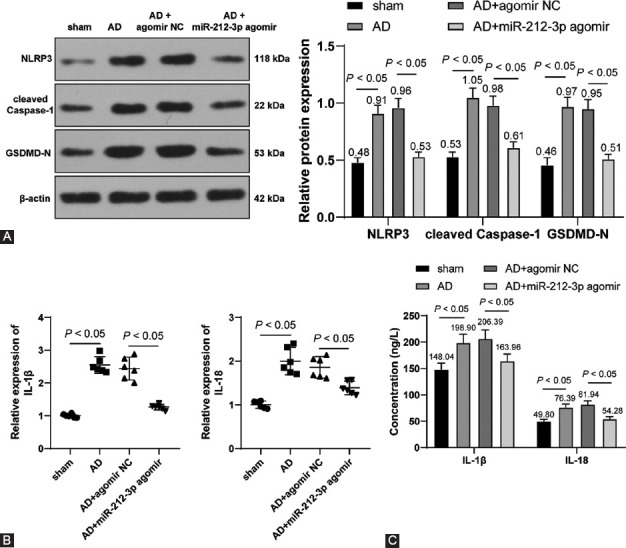
miR-212-3p overexpression suppressed NLRP3/Caspase-1-activated neuronal pyroptosis to attenuate neuroinflammation of AD rats. (A) NLRP3, cleaved Caspase-1, and GSDMD-N expressions were determined through Western blot analysis; IL-1β and IL-18 levels were measured through Western blot analysis (B) and ELISA (C). N=6. Data were formalized as mean ± SD. Data were analyzed using two-way ANOVA, followed by Tukey’s multiple comparison test.

NLRP3 inflammasome plays a critical role in neurodegeneration, such as AD, while the specific blocker of NLRP3 inflammasome could alleviate symptoms of neurodegeneration [10,34]. Therefore, we conjectured that miR-212-3p exerts effects on AD rats through the activation of the NLRP3/Caspase-1 signaling pathway. Our results showed that, compared with the sham group, NLRP3, cleaved Caspase-1, and GSDMD-N expressions were markedly elevated in the hippocampus of AD rats and significantly diminished on the transfection of miR-212-3p agomir (*p* < 0.05, Figure 2A). In addition, RT-qPCR and ELISA results showed that IL-1β and IL-18 levels were elevated in AD rats and declined as a response to miR-212-3p agomir (*p* < 0.05, Figure 2B and C). The above results suggested that miR-212-3p overexpression suppressed NLRP3/Caspase-1-activated neuronal pyroptosis to attenuate neuroinflammation in AD rats.

### miR-212-3p overexpression inhibited NLRP3/Caspase-1-activated neuronal pyroptosis in vitro post-Aβ1-42 treatment

**FIGURE 3:**
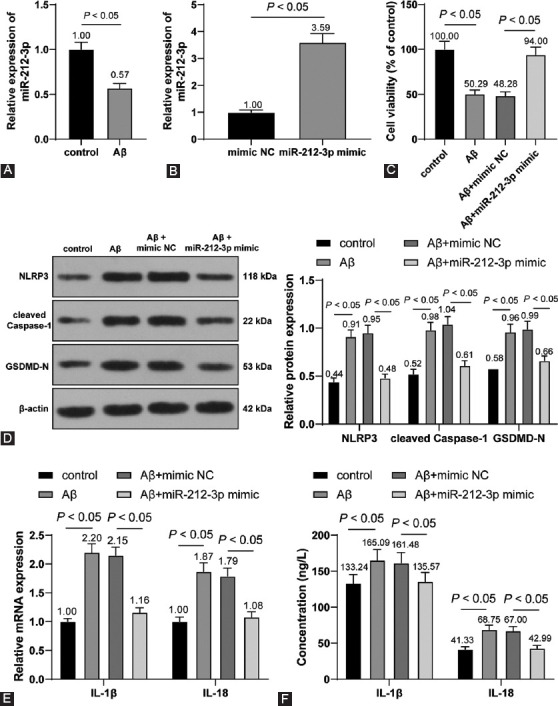
miR-212-3p overexpression inhibited NLRP3/Caspase-1-activated neuronal pyroptosis *in vitro* post-Aβ1-42 treatment. (A) miR-212-3p expression was detected through RT-qPCR after the AD cell model was established using Aβ1-42 treatment with H19-7 cells; (B) miR-212-3p expression was detected through RT-qPCR after transfection of mimic NC and miR-212-3p mimic; (C) cell viability was assessed through the MTT method; (D) NLRP3, cleaved Caspase-1, and GSDMD-N expressions were determined through Western blot analysis; (E and F) IL-1β and IL-18 levels were measured through Western blot analysis (E) and ELISA (F); cell experiments were conducted 3 times. Data were formalized as mean ± SD. Data in Figures A and B were analyzed using T test, data in Figure C were analyzed using one-way ANOVA, and data in Figures D-F were analyzed using two-way ANOVA, followed by Tukey’s multiple comparison test.

The AD cell model was established using H19-7 cells with Aβ1-42 treatment to confirm the role of miR-212-3p in AD *in vitro*. The subsequent RT-qPCR experiments showed that Aβ1-42 markedly inhibited miR-212-3p expression (*p* < 0.05, Figure 3A). To further explore the regulatory role of miR-212-3p in AD *in vitro*, miR-212-3p was overexpressed using miR-212-3p mimic (*p* < 0.05, Figure 3B). It was found that Aβ1-42 treatment reduced the viability of H19-7 cells, while miR-212-3p mimic reversed the inhibitory role of Aβ1-42 in cell viability (*p* < 0.05, Figure 3C).

Moreover, our results showed that Aβ1-42 treatment facilitated NLRP3, cleaved Caspase-1, and GSDMD-N expressions, while miR-212-3p mimic reversed the protein changes (*p* < 0.05, Figure 3D). Besides, IL-1β and IL-18 expressions were augmented by Aβ1-42 treatment and inhibited by miR-212-3p mimic (*p* < 0.05, Figure 3E and F). The above results suggested that miR-212-3p overexpression could inhibit NLRP3/Caspase-1-activated neuronal pyroptosis *in vitro* post-Aβ1-42 treatment.

### miR-212-3p negatively regulated SP1

**FIGURE 4:**
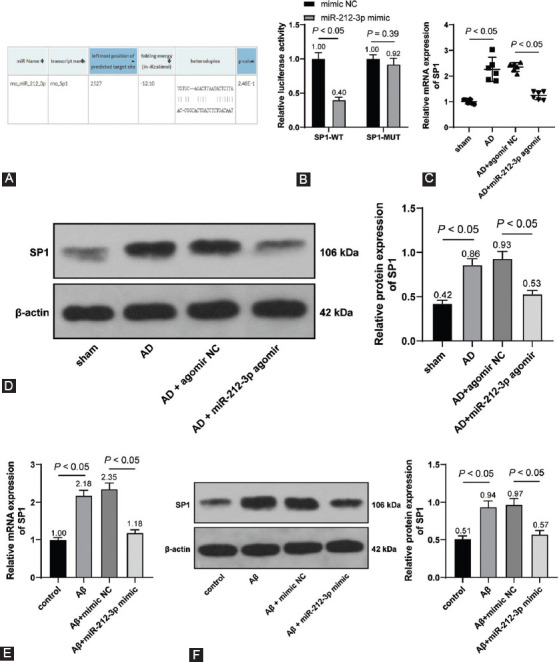
miR-212-3p negatively regulated SP1 expression. (A) The binding of miR-212-3p and SP1 was predicted by the Jefferson website (https://cm.jefferson.edu/rna22/Interactive/RNA22Controller); (B) the binding relationship of miR-212-3p and SP1 was testified dual-luciferase reporter assay; (C and D) SP1 expression in the hippocampus was detected through RT-qPCR (C) and Western blot analysis (D); (E and F) SP1 expression in H19-7 cells was detected through RT-qPCR (E) and Western blot analysis (F). N=6. Cell experiments were repeated 3 times. Data were formalized as mean ± SD. Data in Figure B were analyzed using two-way ANOVA and data in Figures C-F were analyzed using one-way ANOVA, followed by Tukey’s multiple comparison test.

To investigate the downstream mechanism of miR-212-3p in neuroinflammation in AD, the downstream targets of miR-212-3p were predicted through the Jefferson website (https://cm.jefferson.edu/rna22/Interactive/RNA22Controller). Database prediction indicated that miR-212-3p could bind to SP1 (Figure 4A). Accumulating evidence has identified that SP1 is abnormally increased in AD animal models [35]. However, the regulatory mechanism of SP1 remains unclear.

Dual-luciferase reporter assay testified that miR-212-3p could bind to SP1 (*p* < 0.05, Figure 4B). In addition, compared with the sham group, SP1 expression was markedly upregulated in AD rats compared with that in sham-operated rats, and miR-212-3p agomir inhibited SP1 expression (*p* < 0.05, Figure 4C and D). Meanwhile, Aβ1-42 treatment augmented SP1 expression, and miR-212-3p mimic weakened the promoting role of Aβ1-42 in SP1 (*p* < 0.05, Figure 4E and F). The above results suggested that miR-212-3p could negatively regulate SP1 expression.

### SP1 promoted BACE1 transcription

**FIGURE 5:**
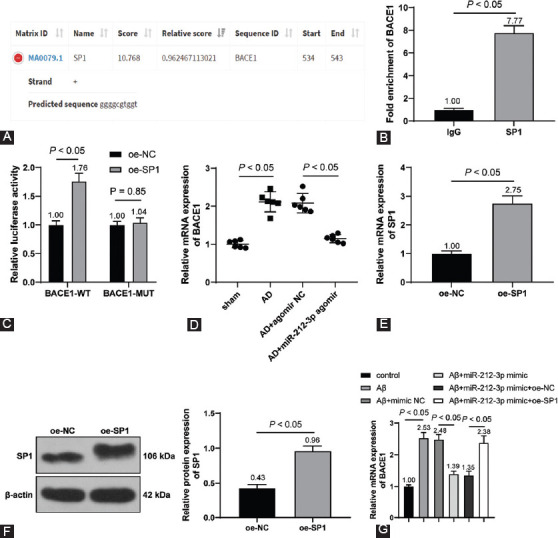
SP1 promoted BACE1 transcription. (A) The binding of SP1 and BACE1 promoter region was predicted through the JASPAR website (http://jaspar.genereg.net/analysis); (B-C) the binding of SP1 and BACE1 promoter region was testified through ChIP (B) and dual-luciferase reporter assay (C); (D) BACE1 expression in the hippocampus was detected through RT-qPCR; (E and F) SP1 expression in cells was determined through RT-qPCR (E) and Western blot analysis (F) on H19-7 cells which were transfected oe-NC and oe-SP1; (G) BACE1 expression in H19-7 cells was determined through RT-qPCR on Aβ1-42-treated H19-7 cells which were transfected with miR-212-3p mimic and oe-SP1. N=6. Cell experiments were conducted 3 times. Data were formalized as mean ± SD. Data in Figures B and D-F were analyzed using T test and data in Figures C and G were analyzed using two-way ANOVA, followed by Tukey’s multiple comparison test.

SP1 serves as a transcription factor to activate the expressions of downstream targets [36]. CNS diseases are associated with the alteration of genetic expressions, including AD. SP1 could regulate the expressions of AD-related genes to participate in the regulation of AD [37]. BACE1 is an Aβ precursor protein lyase and participates in AD progression [38]. Presumably, there is a regulatory relationship between SP1 and BACE1 in AD. The binding relationship of SP1 and the BACE1 promoter region was predicted through the JASPAR website (http://jaspar.genereg.net/analysis) (Figure 5A) and testified through dual-luciferase reporter assay (*p* < 0.05, Figure 5B and C).

Further, we detected BACE1 expression in the hippocampus and found that compared with the sham group, BACE1 expression was markedly upregulated in the AD group; and compared with the AD + agomir NC group, BACE1 expression was markedly downregulated in the AD+miR-212-3p agomir (*p* < 0.05, Figure 5D). After SP1 was overexpressed *in vitro* (*p* < 0.05, Figure 5E and F), Aβ1-42-treated H19-7 cells were simultaneously transfected with miR-212-3p mimic and oe-SP1. Our results showed that Aβ1-42 treatment increased BACE1 transcriptional level, miR-212-3p mimic inhibited BACE1 transcription, and oe-SP1 facilitated BACE1 transcription again (*p* < 0.05, Figure 5G). Briefly, SP1 could promote BACE1 transcription, and miR-212-3p overexpression inhibited SP1 to suppress BACE1 transcription.

### BACE1 upregulation activated the NLRP3/Caspase-1 signaling pathway to reverse the inhibitory role of miR-212-3p overexpression in neuronal pyroptosis

**FIGURE 6:**
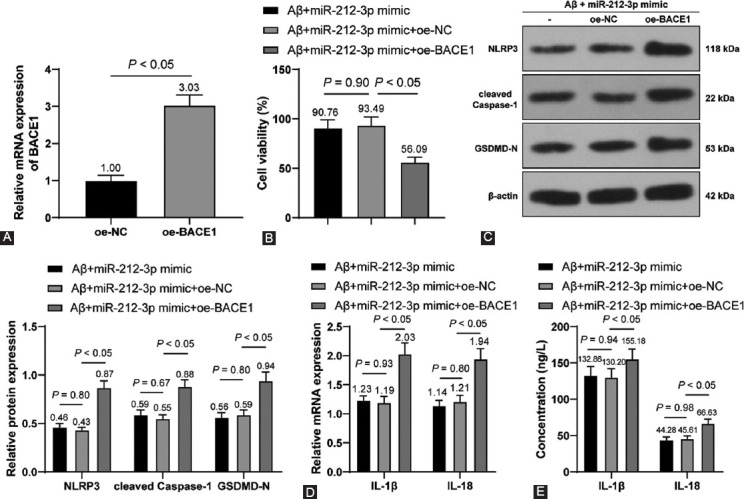
BACE1 upregulation activated NLRP3/Caspase-1 signaling pathway to reverse the inhibitory role of miR-212-3p overexpression in neuronal pyroptosis. (A) BACE1 was detected through RT-qPCR after H19-7 cells were transfected with oe-NC and oe-BACE1; (B) cell viability was assessed through the MTT method after Aβ1-42-treated H19-7 cells were transfected miR-212-3p mimic and oe-BACE1; (C) NLRP3, cleaved Caspase-1, and GSDMD-N expressions were determined through Western blot analysis; (D-E) IL-1β and IL-18 levels were measured through Western blot analysis (D) and ELISA (E). Cell experiments were conducted 3 times. Data were formalized as mean ± SD. Data in Figures A were analyzed using T test, data in Figure B were analyzed using one-way ANOVA, and data in Figures C-E were analyzed using two-way ANOVA, followed by Tukey’s multiple comparison test.

At last, on the acquisition of BACE1 overexpression vector (oe-BACE1) (*p* < 0.05, Figure 6A), Aβ1-42-treated H19-7 cells were transfected with miR-212-3p mimic and oe-BACE1. It was observed that compared with the Aβ + miR-212-3p mimic + oe-NC group, cell viability was markedly weakened in the βb + miR-212-3p mimic+oe-BACE1 (*p* < 0.05, Figure 6B). Moreover, compared with the Ab + miR-212-3p mimic + oe-NC group, NLRP3, cleaved Caspase-1, GSDMD-N, IL-1β, and IL-18 expressions were markedly augmented in the Aβ+miR-212-3p mimic+oe-BACE1 (*p* < 0.05, Figure 6 C-E). Overall, BACE1 upregulation could activate the NLRP3/Caspase-1 signaling pathway to reverse the inhibitory role of miR-212-3p overexpression in neuronal pyroptosis.

## DISCUSSION

**FIGURE 7:**
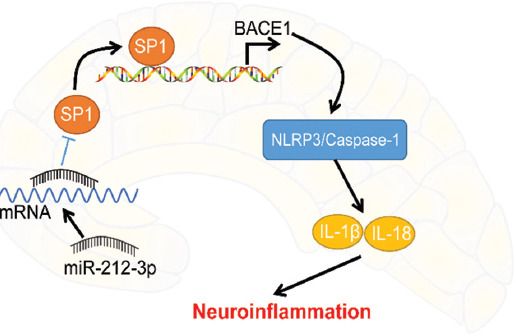
Molecular mechanism of miR-212-3p in AD-induced neuroinflammation. SP1 bounds to the BACE1 promoter region to activate BACE1 expression. miR-212-3p inhibited SP1 and then BACE1 to block the activation of the NLRP3/Caspase-1 signaling pathway, thus attenuating neuroinflammation in AD rats.

AD is manifested as an age-associated decline of cognitive and neurological functions [1]. Neuroinflammation remains a major culprit of AD occurrence, but few targets display reliable effects to solve it in clinical settings [5]. Hard done work of our peers has unveiled that dysregulation of miRNAs is associated with neuroinflammation in AD [39]. In the present study, we highlighted that miR-212-3p inhibited SP1 and BACE1 to block the activation of the NLRP3/Caspase-1 signaling pathway, thus attenuating neuroinflammation in AD rats (Figure 7).

Essentially, miR-212 exerts effects in alleviating neurotoxicity of Aβ protein and tau-phosphorylation in AD [16,17]. Among the miR-212 family members, miR-212-3p was shown to display a dramatic decline in the gray matter of AD patients [40]. To analyze the therapeutic significance of miR-212-3p, we first established an AD rat model using an injection of Aβ1-42 and then overexpressed miR-212-3p in AD rats. Our experiments showed that miR-212-3p overexpression shortened escape latency, while prolonged the time in the target quadrant and the time to pass the target area, revealing that miR-212-3p improved the learning and memory capacities of AD rats. In accordance, miR-212 deficiency in AD mice and humans contributes to tau aggregation and neural dysfunction that lead to memory loss [41,42]. Histologically, miR-212-3p overexpression alleviated neuronal injury manifested in a form of clear neuronal layers and increased numbers of normal neurons with round nucleus and Nissl-positive neurons. In favor of our results, miR-212-3p overexpression could inhibit neuronal loss and oxidative stress in AD [33,40,43]. Altogether, we demonstrated that miR-212-3p overexpression improves the neurological function of AD rats.

NLRP3 inflammasome emerges as a critical player in neuronal inflammation and pyroptosis in AD [10,11]. NLRP3 inflammasome activation cleaved dormant pro-Caspase-1 into activated Caspase-1, further leading to the production of matured IL-1β and IL-18 [44]. Presumably, miR-212-3p acts in AD through regulating the NLRP3/Caspase-1 signaling pathway. Our subsequent experiments showed that miR-212-3p overexpression reduced the expressions of NLRP3, cleaved Caspase-1, GSDMD-N, IL-1β, and IL-18, indicating the properties of miR-212-3p against pyroptosis and inflammation in AD rats.

On top of that, H19-7 cells were treated with Aβ1-42 to establish an AD cell model. Our experiments revealed that miR-212-3p overexpression increased H19-7 cell viability, coincided with reduced levels of pyroptotic and inflammatory cytokines. Consistently, miR-212 depletion facilitates neuronal ferroptosis to induce neurological dysfunction in controlled cortical impact-treated mice [45]. Furthermore, miR-212-3p plays a suppressive role in macrophage activation-induced inflammatory response [46,47]. However, the role of miR-212-3p as a pyroptosis inhibitor has not been reported before. Taken all, we are the first to uncover that miR-212-3p overexpression suppresses NLRP3/Caspase-1-activated neuronal pyroptosis to attenuate neuroinflammation in AD rat and cell models.

Subsequently, we focused on the downstream mechanism of miR-212-3p. Prior research has highlighted that miR-212-3p inhibits both protein and mRNA levels of SP1 in breast cancer cells [18]. SP1 has a dramatic elevation in AD and is associated with cognitive deficits, inflammation, and neuronal survival [35]. The binding relationship of miR-212-3p and SP1 was verified through dual-luciferase reporter assay. SP1 was increased in AD rats and inhibited by miR-212-3p overexpression, indicating the negative correlation between miR-212-3p and SP1. In addition, SP1 acts as a pathogen in AD through activation of BACE1 [22,48]. BACE1 inhibitor serves as an effective therapeutic option to improve neurofunctional in AD [49,50]. We confirmed that SP1 could bind to the BACE1 promoter region using the ChIP and dual-luciferase reporter assays, and observed that BACE1 was increased in AD rats, reduced by miR-212-3p overexpression, and augmented by SP1 overexpression, suggesting that miR-212-3p inhibited SP1 to suppress BACE1 transcription both in AD rats and cells. Afterward, a collaborative experiment was performed to evaluate the functions of BACE1 on AD regulated by miR-212-3p. Our results revealed that BACE1 reversed the suppressive role of miR-212-3p overexpression in neuronal pyroptosis and neuroinflammation. In consistence, BACE1 is positively associated with NLRP3 inflammasome activation and its downregulation restricts the secretion of pro-inflammatory factors and benefit neuronal life cycle in AD [51-53]. Collectively, we initially demonstrated that miR-212-3p attenuates NLRP3/Caspase-1-activated neuronal pyroptosis through inhibiting the SP1/BACE1 axis.

## CONCLUSION

Our findings initially unveiled that miR-212-3p suppresses NLRP3/Caspase-1 signal-induced neuronal pyroptosis through regulating the SP1/BACE1 axis to neuroinflammation in AD and hinted at the role of miR-212-3p as a potential therapeutic and diagnostic target in AD treatment. However, since we just detected miR-212-3p expression in AD cell and animal models, its expression in clinical simple remains unknown, and this study failed to explore the mechanism of miR-212-3p downregulation in AD and evaluate the role of miR-212-3p in AD-induced oxidative stress injury. In the future, we plan to analyze miR-212-3p expression in AD clinical samples to validate its therapeutic and diagnostic values in the clinic. To achieve this goal, we have to overcome challenges, such as the long time span of the clinical study, a wide range of involved people, and various uncontrollable factors.
